# Design, Manufacturing and Acoustic Assessment of Polymer Mouthpieces for Trombones

**DOI:** 10.3390/polym15071667

**Published:** 2023-03-27

**Authors:** Juan C. Rodríguez, Romina del Rey, Miguel A. Peydro, Jesús Alba, Juan L. Gámez

**Affiliations:** 1 Centre for Physics Technologies: Acoustics, Materials and Astrophysics, Universitat Politècnica de València, EPS Gandia, C/Paranimf, 1, 46730 Grao de Gandia, Spain; juarodve@upv.es; 2 Centre for Physics Technologies: Acoustics, Materials and Astrophysics, Universitat Politècnica de València, EPS de Alcoy, Plaza Ferrándiz y Carbonell, 1, 03801 Alcoy, Spain; roderey@fis.upv.es; 3 Institute of Materials Technology, Universitat Politècnica de València, EPS de Alcoy, Plaza Ferrándiz y Carbonell, 1, 03801 Alcoy, Spain; mpeydro@upv.es; 4 Department of Graphics Engineering, Universitat Politècnica de València, EPS de Alcoy, Plaza Ferrándiz y Carbonell, 1, 03801 Alcoy, Spain; jgamez@dig.upv.es

**Keywords:** additive manufacturing, subtractive manufacturing, polymer composites, trombone, mouthpiece

## Abstract

Brass instruments mouthpieces have been historically built using metal materials, usually brass. With the auge of additive manufacturing technologies new possibilities have arisen, both for testing alternative designs and for using new materials. This work assesses the use of polymers for manufacturing trombone mouthpieces, specifically PLA and Nylon. The acoustical behavior of these two mouthpieces has been compared with the obtained from a third one, built from brass. Both additive and subtractive manufacturing techniques were used, and the whole manufacturing process is described. The mouthpieces were acoustically assessed in an anechoic chamber with the collaboration of a professional performer. The harmonic analysis confirmed that all the manufactured mouthpieces respect the harmonic behavior of the instrument. An energy analysis of the harmonics revealed slight differences between the mouthpieces, which implies differences in the timbre of the instrument. Although these subtle differences would not be acceptable when performing with the instrument in an orchestra, they could be perfectly valid for early learners, personal rehearsals or any kind of alternative performance.

## 1. Introduction

The trombone is one of the several instruments from the brass family. Instruments of this family are usually built from metal pieces. That is what distinguishes them from woodwind instruments, the other family of wind instruments, which combine materials like wood and metal.

With the auge of 3D printing technologies, new materials are being incorporated to build parts of musical instruments or even complete instruments [1,2,3,4,5].

The use of additive manufacturing techniques presents some advantages over traditional machining. Regarding material waste, since 3D printing techniques use almost only the necessary material for the manufacturing of the piece, the material waste is minimized, unlike in a machining process, which produces a significant amount of waste in the form of chips, which later can be recycled but implies an additional stage in the manufacturing process. In the case of complex shapes, since with 3D printing, the fabrication is usually done layer by layer, it is possible to achieve very complex shapes that would be only achievable by experts, or sometimes even impossible to manufacture, with traditional machining. This could be the case of non-straight perforations, for example. This makes it possible for people not qualified for manual manufacturing but with some knowledge about 3D-printing processes to transform complex designs into usable pieces of controlled properties since human intervention is almost unnecessary after the 3D CAD design is finished, and allows testing the designs in an early stage quickly. Finally, there is a lower manufacturing cost. With 3D printing, parts are obtained quickly and cheaply since 3D printers are inexpensive, and only the necessary material is used. The cost of the Artillery Genius 3D printer used in this work is around 300 €. On the other hand, machining on CNC lathes is much more expensive, mainly due to the acquisition cost of the machinery, which in the case of the Pinacho ST 225 × 1000 CNC lathe, also used in this work, was around 25k €. Considering that in the case of customized wind instrument mouthpieces, several prototypes can be needed to completely adapt them to the performer’s needs, the possibility of quick and low-cost manufacturing after each design modification motivates the assessment of 3D printing technologies for mouthpiece manufacturing.

Despite the cited advantages of additive manufacturing, limitations like the types of available materials or the durability of the pieces -depending on the type of impression technology and design of the piece- still position these techniques at a disadvantage in front of subtractive manufacturing techniques. Subtractive techniques allow using almost any material at the cost of requiring specific and expensive instrumentation and specialized operators with craft skills for the manufacturing process.

When designing musical instruments, it is essential to understand both the physics underlying sound generation with each instrument and the mechanisms of human sound perception. These topics have been widely studied and covered in the available literature [6,7,8,9,10] and also specifically for brass instruments [11,12,13,14].

In the case of trombones, the physics governing their behavior considers the different parts of the instrument: the mouthpiece, the cylindrical tube, and the bell. All of them influence the final sound generated by the instrument when the player blows the mouthpiece. The effects of materials and the shape of the different parts of the instruments on the generated sound have been analyzed in several studies [15,16,17,18].

**Figure 1:**
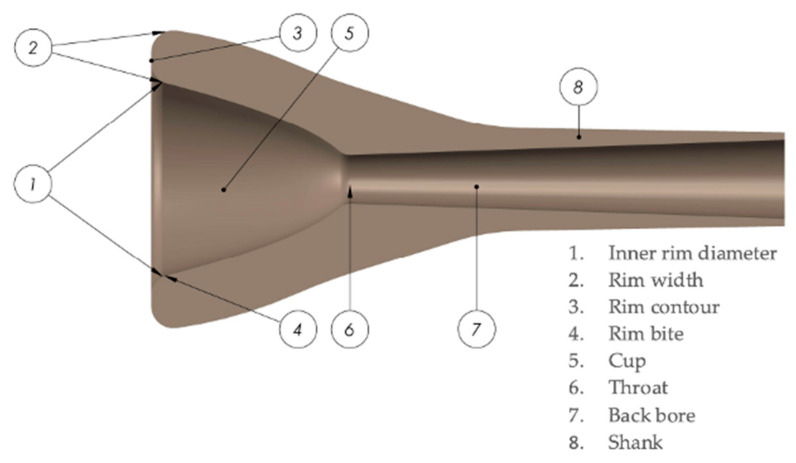
Parts of a trombone mouthpiece.

Several parts must be considered when analyzing the mouthpiece behavior and comparing different designs. Figure 1 details the different parts of a trombone mouthpiece.

Commercial mouthpieces are mainly made of brass. This material provides several characteristics which are crucial for mouthpieces, such as controlled density and durability against corrosion caused by the interpreter’s saliva,

The cited properties can also be found in other materials, which opens the door to analyzing their acoustic behavior compared to traditional materials and even more considering that some players present allergies to brass. Others may want to explore different chromas and incorporate them into their sound. And finally, considering the economic aspect also opens the door to trying cheaper materials, especially between initial learners.

Some studies analyzing the acoustic behavior of 3D-printed mouthpieces have already been published for trombones and other brass and woodwind instruments. Among them, it is worth mentioning the study carried out by Bacciaglia et al. [19], where an end-user-oriented approach was used to manufacture several mouthpiece designs, which were 3D printed in polylactic acid (PLA) and then scored by user feedback. It is also worth mentioning the work carried out by Tarrazó-Serrano et al. in [20], in which clones of two commercial trumpet mouthpieces were 3D-printed in PLA and, after conducting some recordings, the results were assessed by comparing the spectral content. Both studies conclude that the use of 3D-printing technologies can be useful for providing alternatives to musicians, which motivated the present work where an acoustical assessment of trombone mouthpieces built from different materials, using both additive and subtractive manufacturing techniques, is carried out.

The following sections will present the design, manufacturing, and acoustic assessment of three mouthpieces made with polymers and metal materials. All the stages of the manufacturing process and the methodology used to assess the manufactured mouthpieces will be described in detail.

The obtained results during the acoustic tests suggest the feasibility of using polymers for manufacturing brass instrument mouthpieces.

## 2. Materials and Methods

### 2.1. Materials

When selecting alternative materials for mouthpieces, some technical requirements should be met. The following criteria were applied to filter the materials for this study:

- Physical properties: the mouthpiece should not be too heavy as otherwise; it would not be comfortable for the performer. This makes the density of the material a critical aspect to consider.
- Magnetic properties: avoiding the accumulation of magnetic particles in the mouthpiece is essential. This will preserve the hygiene of the mouthpiece.
- Optical properties: when playing a brass instrument, a large amount of water vapor and saliva is released. On an aesthetic level, it is convenient that the mouthpiece is opaque.
- Durability: again, due to the constant contact with fluids, it is convenient that the material has excellent behavior against salt and fresh water, that is: non-corrosive.
- Mechanizability: since the mouthpieces have complex shapes, the material must be relatively easy to mechanize.
- Other non-technical criteria that were also considered are:
- Material family: Since the study’s objective is to assess the behavior of polymeric materials and the most common material found in commercial mouthpieces is brass, the search was also restricted to polymeric and metal materials.
- Price: again, since brass is the most common material for mouthpieces, selected materials should not be more expensive than brass.

Considering technical and not technical criteria, the final filters that were applied for the initial search are described in Table 1.

With the selected criteria, an initial search was conducted using Granta EduPack software [21]. From the resulting list of materials, two polymers were chosen for the study: Polylactic acid (PLA) and Nylon (PA-6). Besides them, brass was also used in the study to compare the most commercially used material for mouthpieces with the two selected polymers. The properties of the selected materials are presented in Table 2.

### 2.2. Design and Manufacturing of the Mouthpieces

**Figure 2:**
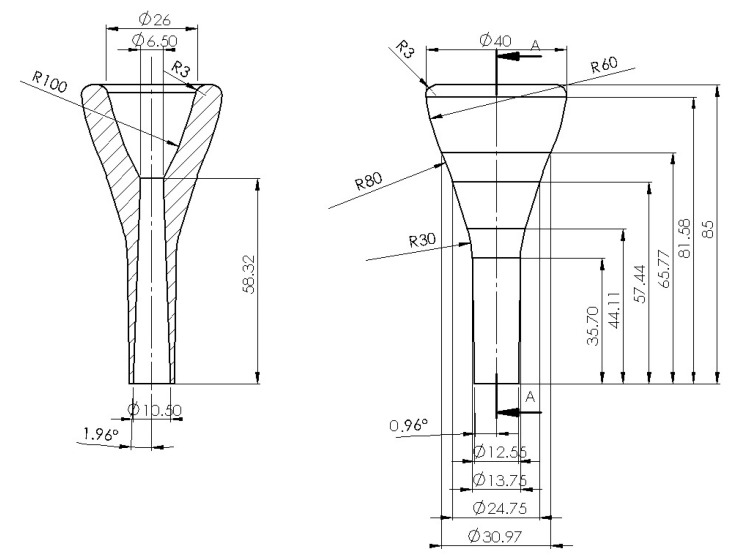
Dimensions of the designed mouthpiece.

The mouthpiece used in this work was designed taking a commonly used commercial model as a reference but adapting it to the preferences of the trombone player that collaborated in the acoustic tests. The designed mouthpiece has a U-form cup, and the ring diameter is slightly smaller than the original design. The final dimensions of the mouthpiece are presented in Figure 2.

**Figure 3:**
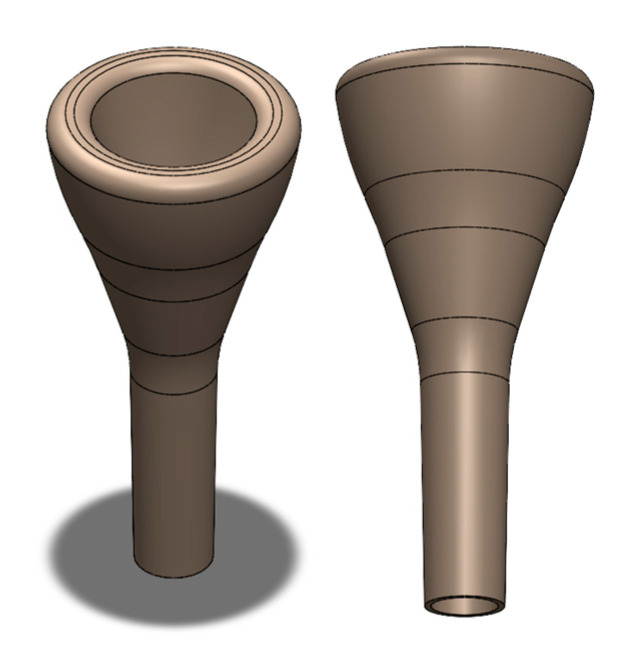
3D CAD model of the mouthpiece.

It should be taken into account that the main objective of the work was to compare different materials with the same mouthpiece design. In no way was it intended to design an innovative mouthpiece. The final 3D CAD model of the designed mouthpiece is presented in Figure 3.

Once the 3D CAD design was finished, the manufacturing process started. All the mouthpieces used in this work were specifically manufactured for this purpose, using both additive and subtractive manufacturing techniques.

**Figure 4:**
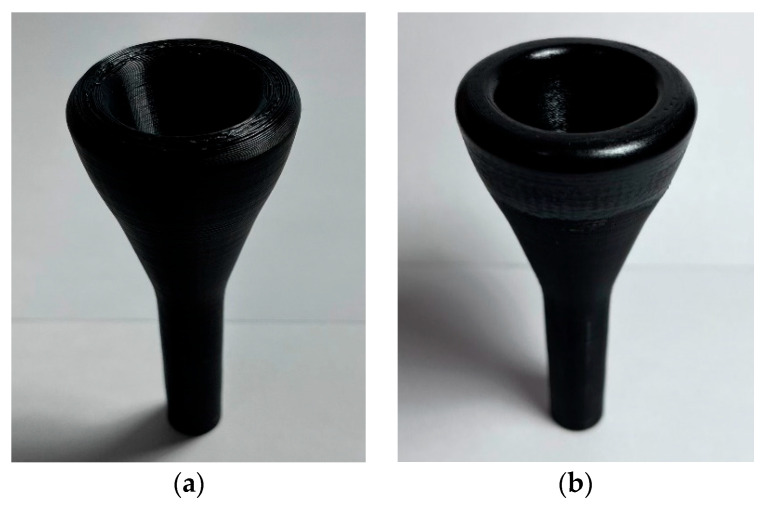
3D Printed mouthpiece: (**a**) just after printing; (**b**) finished mouthpiece after manual sanding of the rim.

In the case of additive manufacturing, the 3D CAD model was prepared for printing with the software Ultimaker Cura 4.13, and printed using an Artillery Genius 3D printer, with PLA as printing material and the settings detailed in Table 3. As seen in Figure 4, the ring of the mouthpiece was manually smoothed after printing.

**Figure 5:**
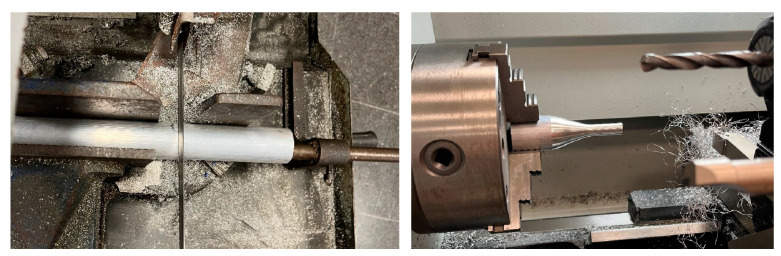
Cutting of the billet and external shaping of the mouthpieces on a Pinacho ST 225 × 1000 CNC lathe.

In the case of the subtractive manufactured mouthpieces, the process always started with a cylindrical billet of material, as presented in Figure 5, and with the help of several tools -such as lathes and saws- the designed mouthpiece was manufactured in three different materials: nylon, aluminum, and brass. However, for this work, the behavior of the aluminum mouthpiece has not been analyzed, since only those made with polymers have been compared to the brass one.

To achieve the internal and external shape of the designed mouthpiece, the manufacturing process consisted of the following stages:

1. Tracer cutting of the billet.
2. Drilling of each section of the mouthpiece.
3. Polishing the interior of the mouthpiece.
4. Finishing of the edges.
5. Conical machining of the shank.
6. Polishing of the whole part.

Different tools were used during manufacturing, including manual and CNC lathes, band saws and tracers, and sandpaper.

Figure 5 shows the process carried out to shape the exterior of the mouthpieces from a cylindrical billet of each material. For this, the use was made of a computer numerical control (CNC) lathe. The CNC code was made with the WinUnisoft software [22], with the ISO language of the control Fagor 8055, with the following cutting conditions: cutting speed: 200 m/min, depth of cut: 1 mm and feed: 0.1 mm/rev.

**Figure 6:**
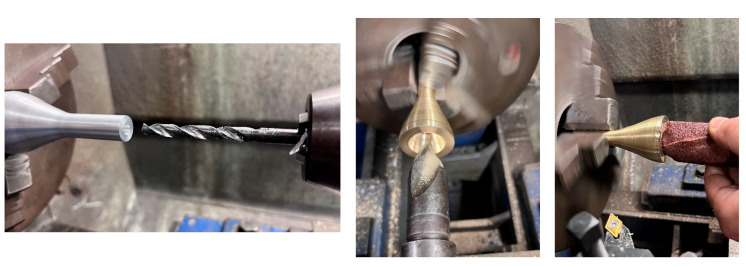
Internal drilling and sanding of the throat on a Pinacho S-90/200 manual lathe.

The backbore of the mouthpieces was drilled with the help of a manual lathe with a digital display that allowed the process to be precisely controlled. After cutting off the excess material used to hold the mouthpiece in the lathe, it was time to shape the interior of the cup. A custom-made drill bit was used for this purpose. Figure 6 shows some images of the drilling and sanding processes.

The mouthpiece is placed on a trombone clamping. This can be done because the shank of the mouthpiece is wedge-shaped with an angle of 1°.

**Figure 7:**
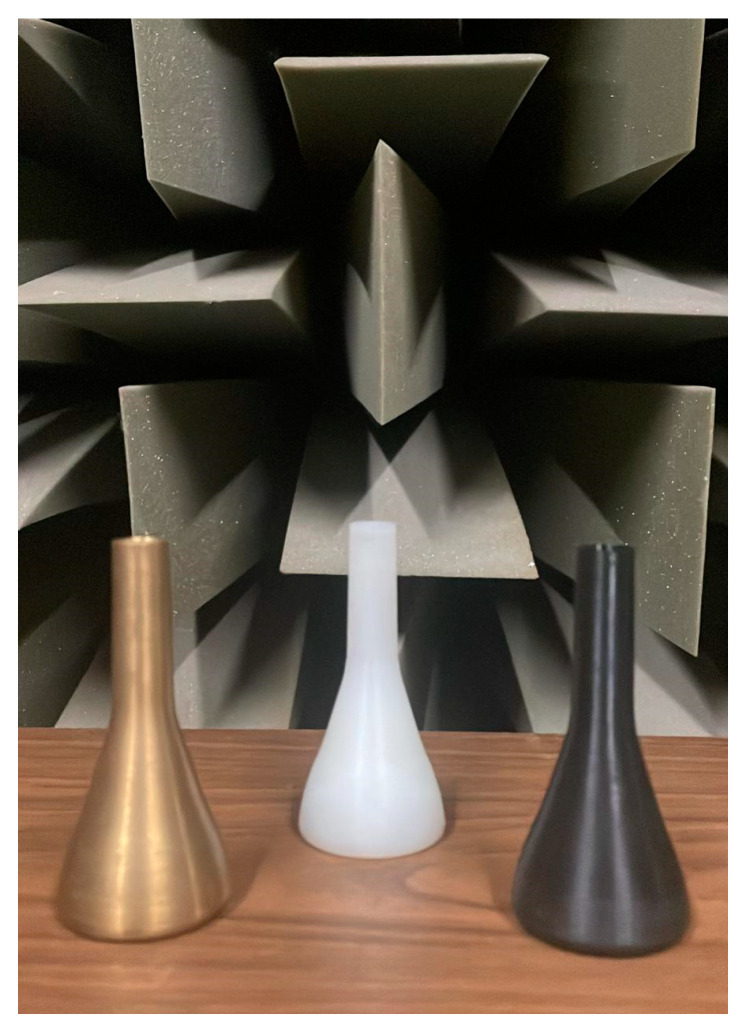
Manufactured mouthpieces in the anechoic chamber of the Higher Polytechnic School of Gandia. Materials, from left to right: brass, nylon and PLA.

The manufactured mouthpieces can be seen in Figure 7. As observed, the same shape was achieved with additive and subtractive manufacturing processes.

### 2.3. Analysis of the Harmonic Response of the Instrument

A brief introduction to the physics of brass instruments is required to understand the analysis of the acoustic response of the manufactured mouthpieces. When playing brass instruments carefully, a sustained tone brings an almost perfectly periodic signal with several harmonics. That implies that deviations from that ideal response can be attributed to the performer playing the instrument [8].

Analyzing the frequency response is a common practice when studying the behavior of a musical instrument [13,23,24,25], comparing different models or the differences between performers.

The trombone’s sound harmonic structure comes from the instrument’s tube nature. The trombone can be seen as a folded hollow tube with a mouthpiece at one end and a bell at the other. The performer closes the mouthpiece end of the instrument, leaving the other end of the tube open with a bell termination. This gives a harmonic series formed by the fundamental note and overtones that satisfy Equation (1).

(1) $$\overset{\infty }{\underset{n=1}{\sum }}nf_{1}=1f_{1}+2f_{1}+3f_{1}+4f_{1}+\cdots +nf_{1},$$

where n is a natural number.

When choosing some of the most significative playable notes of the instrument for the analysis, it is essential to introduce the concept of tessitura briefly. The tessitura of an instrument determines the range of notes that the instrument can reproduce, i.e., the frequency range of the fundamental notes than can be produced with the instrument.

Five different types of trombones exist with different tessituras: the soprano trombone, the alto trombone, the tenor trombone, the bass trombone, and the contrabass trombone. This study focuses on the tenor trombone, which tessitura ranges from notes E2 (excluding fundamentals or pedal notes) to F5, corresponding to 82 Hz and 698.46 Hz frequencies, respectively, considering a frequency of 440 Hz for A4.

After consulting several professional musicians, a consensus was reached to analyze the sound of the trombone in much of its range, so the range from A2 to A4 was selected for the tests.

The tests were conducted in the anechoic chamber of the Higher Polytechnic School of Gandia with a Brüel & Kjaer Type 2270 Sound Level Meter with the Fast Fourier Transform (FFT) application. The tests were conducted with the help of a professional performer to minimize human error when playing the instrument so that any possible difference could be attributed to the mouthpieces. Three takes of each note with each of the mouthpieces were taken and the average of them was used to compare the acoustical behavior of the mouthpieces.

## 3. Results

### Harmonic Analysis

The results of the tests conducted in collaboration with a professional performer have been divided into three sections based on the register of the played notes. Three notes for each register have been analyzed, grouped as follows:

- Bass register: F2, G2, and A2 musical notes.
- Mid register: E3, F3, and G3 musical notes.
- Treble register: D4, E4, and F4 musical notes.

**Figure 8:**
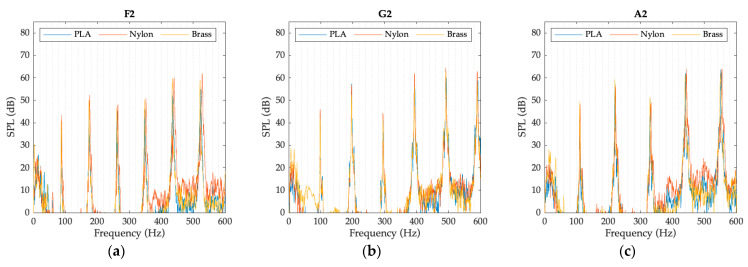
Bass register spectrum for each mouthpiece: (**a**) F2 note; (**b**) G2 note; (**c**) A2 note.

**Figure 9:**
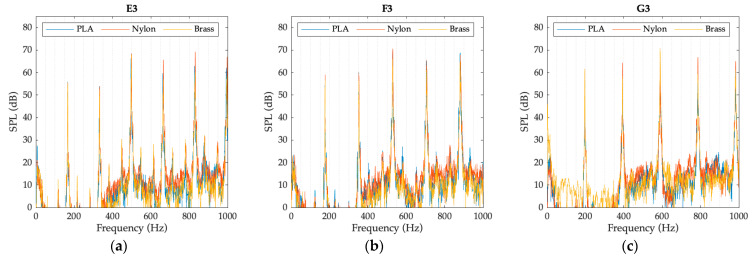
Mid register spectrum for each mouthpiece: (**a**) E3 note; (**b**) F3 note; (**c**) G3 note.

**Figure 10:**
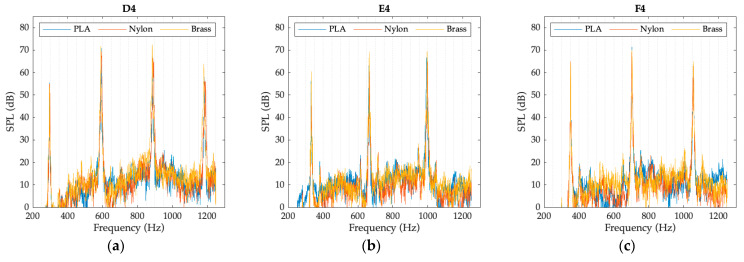
High register spectrum for each mouthpiece: (**a**) D4 note; (**b**) E4 note; (**c**) F4 note.

The graphs presented in Figure 8, Figure 9 and Figure 10 represent the spectrum of the notes corresponding to each register. The sound pressure level (SPL), obtained with each of the mouthpieces when playing the selected notes, is plotted as a function of frequency. The graphs clearly show the fundamental note and some of the harmonics of its harmonic series.

As perceived in the previous analysis, there is practically no difference between the different mouthpieces in the frequencies of the tones and their harmonics. The standard deviation between frequencies has been calculated using Equation (2), with values between 0% and 0.6%.

(2) $$\sigma =\sqrt{\frac{\sum_{i=1}^{N}\left(x_{i}-\bar{x}\right)}{N}},$$

where *N* is the number of mouthpieces, $x_{i}$ correspond to the frequency of the harmonic obtained with each of the mouthpieces and $\bar{x}$ is the mean frequency value of the harmonic for the *N* mouthpieces. The deviation between frequencies was calculated for each harmonic of each note.

**Figure 11:**
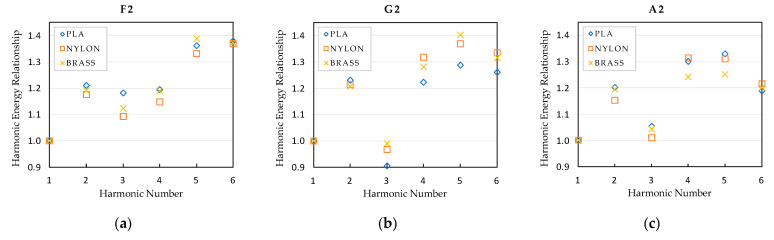
Harmonic energy relationship of the bass register notes: (**a**) E2 note; (**b**) F2 note; (**c**) G2 note.

**Figure 12:**
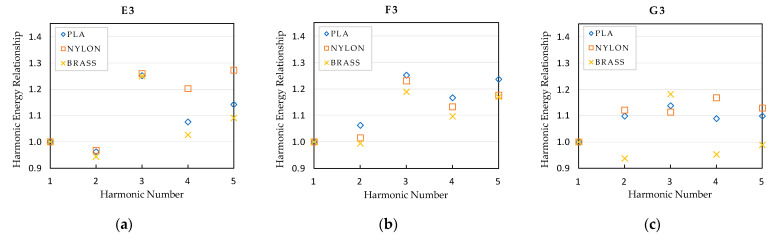
Harmonic energy relationship of the mid register notes: (**a**) E3 note; (**b**) F3 note; (**c**) G3 note.

**Figure 13:**
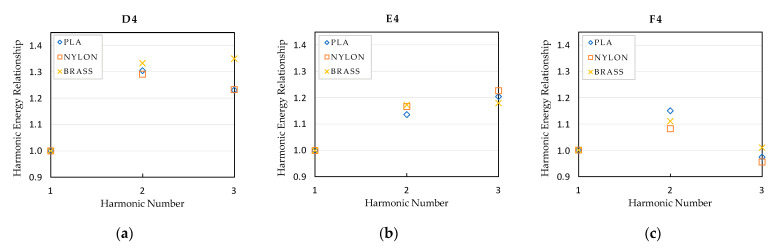
Harmonic energy relationship of the high register notes: (**a**) F2 note; (**b**) G2 note; (**c**) A2 note.

Besides analyzing the frequency of the tones and harmonics, their amplitude has also been analyzed. In order to eliminate the human effect due to possible variations of the performer while playing the notes with each mouthpiece, the spectrums have been normalized taking the fundamental note as main reference. Graphics presented in Figure 11, Figure 12 and Figure 13 represent the relationship between the sound pressure level of each harmonic and the fundamental note, that is, the first harmonic.

As observed in previous figures, there are subtle differences in the amplitude of the harmonics, depending on the type of mouthpiece, but their tendency is practically the same in all cases, except in note G3, where the harmonic energy relationship of the brass mouthpiece is different to that obtained with the nylon and PLA mouthpieces. At this point, it is essential to take into account the properties of the materials presented in Table 2, where significant differences in the values for the density and the Young modulus of the materials can be observed between the polymers and the brass materials. A deep analysis of the effect of these properties on the resonant frequencies of the mouthpieces is required to objectively establish a relationship between these values and the differences detected in the harmonic response. In the case of the polymer mouthpieces, it also has to be taken into account that the infill percentage used for printing the PLA mouthpiece is also probably causing slight differences in the amplitude of the harmonics with respect to the nylon one.

The differences in the energy level of the harmonics affect the timbre of the instrument, so the sound of the instrument slightly varies when using each one of the tested mouthpieces. Depending on the expertise level of the performer, these differences will hardly be noticeable or may represent a clear criterion for selecting one mouthpiece or another.

## 4. Discussion

The complete design and manufacturing process of three different trombone mouthpieces has been described. The acoustic performance of two polymer materials has been evaluated against brass, the most commonly used material for trombone mouthpiece manufacturing. Both additive and subtractive manufacturing techniques were used, and the processes have been described in detail.

The three mouthpieces were subjected to acoustic tests, carried out with the collaboration of a professional musician, to minimize uncertainty due to possible human errors during the reproduction of the analyzed musical notes.

The harmonic analysis of the responses obtained during the tests showed that the three mouthpieces kept the expected harmonic response of the instrument, with slight variations that never exceeded 0.5% of the expected frequency. In the case of the energy level of the harmonics, some differences have been observed between the mouthpieces. This implies a slight difference in the instrument’s timbre that would not be acceptable in classical music performances, where the classical trombone sound is expected, but for early learners, individual rehearsals, or experimental showcases, these timbre differences should not be a problem.

The obtained results open the door to considering polymer materials as a feasible alternative for some uses. The assessed materials or other polymers could be tested until finding the one that fits the performer’s needs. It must be taken into account that some performers present allergies to brass, so these types of materials could be a good alternative.

Further studies are still to be conducted, so the effect of coatings to reduce the roughness and minimize the accumulation of particles in the mouthpieces, the use of antibacterial PLA to minimize possible infections with pathogens, or the use of foamed polymers for the 3D printed designs [26,27] to manufacture even lighter mouthpieces can be evaluated.

In any case, the results proved that all the mouthpieces performed well enough to be used with the instrument, at least for rehearsal purposes. This has several advantages, such as the possibility of using a much lighter mouthpiece during rehearsals or designing custom mouthpieces that perfectly adapt to the performer’s needs, with the possibility of testing them quickly and refining them until getting the desired design.

Considering the benefits the polymer mouthpieces provide, their use may be considered by performers in certain situations.

## Figures and Tables

**Table 1:** Defined criteria for material filtering.

Criteria	Desired Value
Physical properties	Density below 9000 kg/m^3^
Magnetic properties	Non-magnetic
Optical properties	Opaque
Durability	Excellent behavior against moisture
Price	Lower than brass

**Table 2:** Structural, mechanical, and thermal properties of the selected materials. Values obtained from Granta EduPack.

Material	Polylactic Acid (PLA)	Nylon (PA-6)	Brass (C-6440)
Density (kg/m^3^)	1240	1120–1150	8400
Young modulus (GPa)	3.3–3.6	1.3–1.6	93
Poisson Coefficient	0.38–0.40	0.34–0.36	0.35
Elastic limit (MPa)	55–72	40–50	138
Resistance to traction (MPa)	47–70	51–62	413
Elongation (%)	3–6	41–59	30
Melting point (°C)	145–177	227–238	890
Glass transition temperature (°C)	52–60	44–56	-
Thermal conductivity (W/m °C)	0.13–0.16	0.26–0.27	121
Specific heat (J/kg °C)	1180–1210	1590–1650	380

**Table 3:** Parameters used for the 3D-printing of the PLA mouthpiece.

Category	Parameter	Value	Category	Parameter	Value
Quality	Layer Height	0.12 mm	Speed	Print Speed	60 mm/s
	Line Width	0.44 mm		Infill Speed	60 mm/s
	Initial Layer Line Width	100%		Wall Speed	30 mm/s
				Travel Speed	150 mm/s
Walls	Wall Thickness	1.32 mm		Initial Layer Print Speed	20 mm/s
	Wall Line Count	3		Initial Layer Travel Speed	100 mm/s
				Skirt/Brim speed	20 mm/s
Top/Bottom	Top/Bottom Thickness	0.84 mm		Number of Slower Layers	2
	Skin Overlap percentage	10%			
	Skin Removal Width	1.32 mm	Travel	Retraction Distance	2 mm
	Skin Expand Width	1.32 mm		Retraction Speed	25 mm/s
Infill	Infill Density	30%	Cooling	Fan Speed	100%
	Infill Line Distance	0.44 mm		Regular Fan Speed at Height	0.36 mm
	Infill Pattern	Cubic			
	Infill Overlap Percentage	30%	Build Plate Adhesion	Support	Not
	Infill Layer Thickness	0.12 mm		Build Plate Adhesion	Skirt
				Skirt line Count	4
Material (PLA)	Printing Temperature	200 °C		Skirt Distance	10 mm
	Build Plate Temperature	60 °C		Skirt/Brim minimum Length	250 mm
	Flow	100%			

## Data Availability

The data presented in this study are available on request from the corresponding author.
